# Patients' Perceptions of Nurses' Behaviour That Influence Patient Participation in Nursing Care: A Critical Incident Study

**DOI:** 10.1155/2011/534060

**Published:** 2011-04-27

**Authors:** Inga E. Larsson, Monika J. M. Sahlsten, Kerstin Segesten, Kaety A. E. Plos

**Affiliations:** ^1^Department of Nursing, Health and Culture, University West, 461 86 Trollhättan, Sweden; ^2^School of Life Sciences, University of Skövde, Högskolevägen 1, 541 28 Skövde, Sweden; ^3^Institute of Health and Care Sciences, University College of Borås, Allégatan 1, 501 90 Borås, Sweden; ^4^Institute of Health and Care Sciences, The Sahlgrenska Academy at Gothenburg University, Box 457, 405 30 Gothenburg, Sweden

## Abstract

Patient participation is an important basis for nursing care and medical treatment and is a legal right in many Western countries. Studies have established that patients consider participation to be both obvious and important, but there are also findings showing the opposite and patients often prefer a passive recipient role. Knowledge of what may influence patients' participation is thus of great importance. The aim was to identify incidents and nurses' behaviours that influence patients' participation in nursing care based on patients' experiences from inpatient somatic care. The Critical Incident Technique (CIT) was employed. Interviews were performed with patients (*n* = 17), recruited from somatic inpatient care at an internal medical clinic in West Sweden. This study provided a picture of incidents, nurses' behaviours that stimulate or inhibit patients' participation, and patient reactions on nurses' behaviours. Incidents took place during medical ward round, nursing ward round, information session, nursing documentation, drug administration, and meal.

## 1. Introduction

Patients' active participation in their own care is known to increase motivation and adherence to prescriptions, give better treatment results, create greater satisfaction with received care [1], and reduce stress and anxiety [2]. Patient participation is an important basis for nursing care and medical treatment and it is also a legal right in many Western countries. Studies have established that patients consider participation to be both obvious and important [3, 4], but there are also findings showing the opposite [5] and patients may prefer a passive recipient role [6, 7]. Knowledge of what may influence patients' participation is thus of great importance when it comes to meeting their expectations and demands.

Previous research focusing on patient participation from a patient perspective has been performed primarily in medicine and is carried out by physicians [8, 9]. Research on patient participation in nursing care has defined participation in performing clinical or daily living skills [10]. Patient participation has been explored in different situations, for example, discharge planning [11–14] and bedside reporting [15] in emergency care [16] and has primarily focused on decision-making in treatment/care (e.g., [17–20]). 

Although nursing theories emphasise participation (e.g., [21]) and studies have explored patient participation in different contexts and situations, there have not been congruence regarding definition, elements, and processes [8, 22, 23]. The lack of clarity is amplified by the use of several terms: patient/client/consumer/user involvement, collaboration, partnership, and influence [8, 17]. However, when the focus is on the patient perspective, the concept of patient participation is commonly used.

Empirical studies have identified conditions for patient participation. Sainio et al. [17] found that the patient needs to have the intellectual ability to understand and choose between alternatives and make decisions about their own nursing care and the nurse must provide adequate and correct information. Tutton [24] emphasized the significance of developing a relationship between nurse and patient and the importance of understanding the patient as well as gaining and retaining an emotional connection. According to Sahlsten et al. [25], a nurse needs to use strategies including building close co-operation with the patient, getting to know the person, and reinforcing self-care capacity. 

Factors restricting participation were identified by Wellard et al. [20]: limited communication between nurses and patients, task-oriented nursing labour, and environmental constraints limiting patients' privacy. Eldh et al. [26] found nonparticipation; when patients lack an equal relationship, respect, and information. According to Efraimsson et al. [12], nonparticipation, occurs when professionals are not attuned to the concerns of the patient and individual needs and when they literally silence or disregard the patient's wishes. Sahlsten et al. [27] found that a nurse can lack theoretical or practical knowledge required as well as an insight that patient participation requires deliberate and planned interaction between nurse and patient together with adjusted actions within every encounter. Larsson et al. [28] recently presented barriers for participation from a patient perspective: facing own inability, meeting lack of empathy, meeting a paternalistic attitude, and sensing structural barriers.

While several studies have addressed patient participation, few accounts exist based on patients' descriptions of decisive incidents that influenced their participation in nursing care. Accordingly, there is a need to explore situations related to critical incidents that influence patient participation. The aim of this study was to identify incidents and nurses' behaviours that influence patients' participation in nursing care based on patients' experiences from inpatient somatic care.

## 2. Method

This study is part of a larger project regarding patient participation in nursing care from the perspective of both patient and nurse. A qualitative approach, using the Critical Incident Technique (CIT), was employed. The CIT is a systematic, inductive, and flexible method where specific descriptions of human behaviour in defined situations are collected [29]. The method is useful in solving practical problems. The central concept in CIT is a critical incident which is a maior event of great importance to the person involved. The incidents are mostly collected in semistructured face-to-face interviews [30], the most satisfactory data collection method in CIT for insuring that all the necessary details are supplied [31]. The informants are asked to provide descriptions of specific incidents, positive and/or negative, which they perceive as significant. Here, these descriptions were collected within the framework of the interview method in order to generate an adequate depth of response. The number of incidents required depends on the complexity of the problem under investigation. It is usually sufficient to collect a total of 100 incidents for a qualitative analysis [29]. 

### 2.1. Informants

The participants (*n* = 17) in this study were recruited from somatic inpatient care. The selection was purposeful. The intention was to have a range of informants able to contribute their experience as patients. The informants were ambulatory patients from three internal medical wards with neither an explicit care philosophy emphasising patient participation, nor a focus on nurse-patient continuity. The wards were focused on (i) stroke, (ii) disorder of kidney and heart, and (iii) lung. All informants were able to communicate in Swedish and had no physical or cognitive deficits hampering the ability to describe their experiences as patients. The time spent on the ward varied from 4 to 19 days. Eight men and nine women participated. Their ages ranged 28–91 years.

### 2.2. Data Collection

 Data were collected by means of semistructured interviews. Nursing care was explained as the interplay with Registered Nurses. The interviewer assisted the patients to describe the specific incidents that have influenced their participation in nursing care. The interview guide consisted of the following questions: describe a positive significant incident which was successful for your participation in your own nursing care, and describe a negative significant incident where you felt nonparticipation. After the patient had identified an event, the following questions, earlier used by Kemppainen [31], were asked: what were the circumstances leading to that event?, exactly what did the nurse do?, how did you respond to the nurse?, and how did the nurse's actions affect your behaviour?. The same wording in the questions was kept throughout all interviews, as recommended by Flanagan [29].

The informants were recruited from an internal medical clinic in a central hospital in West Sweden. Written permission was obtained from the head of the clinic. The head nurse of each ward was contacted by telephone and given information. All the nurses on the selected wards were sent written information regarding aim and procedure. The nurses were asked to approach patients the day before an interview was scheduled and ask whether they were interested in participating in the study or not. Verbal and written information was given to those willing to participate. On the morning of a planned interview, written informed consent was obtained. The interviews were held in the patient's own room or adjacent to the wards in a place where there would be no interruption in order to provide a relaxed environment. Each interview was conducted in an open, friendly atmosphere by the main nurse researcher and lasted between 30 and 60 minutes. Each interview was audio-taped and transcribed verbatim by the main researcher (Inga E. Larsson).

### 2.3. Ethical Issues and Approval

The ethics of scientific work was followed. Each study participant gave his/her written consent after verbal and written information. The Ethics Committee of Gothenburg approved the study (no. 176-06).

### 2.4. Data Analysis

The data material was read repeatedly to obtain a sense of the whole. In the data reduction process, the first step was to identify and mark critical incidents. An incident, either negative or positive, was identified as critical if it was related to the aim of the study and based on a detailed and discernible narrative of a course of events with a distinct start and end. In the data material, a total of 105 critical incidents were identified. Each informant provided between two and 31 incidents. In line with the CIT tradition [29], the classification started with identification and extraction of the incidents. These were analysed, without consideration whether positive or negative, to find similarities. In the second step of analysis, positive and negative incidents were identified as two main areas. Different kinds of nurse behaviours were next identified and classified, followed by patients' responses of these behaviours. Early in the analysis, the number of nurse behaviours increased rapidly and the last three interviews resulted in no new behaviours. To increase credibility, the classifications were discussed by the researchers (Inga E. Larsson, Monika J. M. Sahlsten) as no coassessor was involved in the coding. In addition, two researchers (Kerstin Segesten, Kaety A. E. Plos) not earlier involved in the study examined the classifications including direct quotations. A few clarifications were then made. This final classification system consisted of two main areas and 16 nurse behaviours allocated to six patient responses.

## 3. Results

### 3.1. Incidents

The incidents arise in everyday situations and illuminate both positive and negative turning points (Table 1). They mirror different situations in encounters between patient and nurse. The most frequently described incidents concern situations during medical ward round where the nurse provides no support for patient input, and examples are also given of no preparation ahead of the round. Other incidents concern situations during nursing ward round describing genuine interest and search for patients' experience and views but examples are also given of distance with limited support for patient input. Incidents also describe situations during information session where the nurse provides meaningful and sufficient information but there are also descriptions of missing, insufficient, or inadequate information. The incidents concerning nursing documentation include descriptions of no invitation to participate and examples are also given of no recording of the patients' views. Other incidents concern situations during drug administration where the nurse leaves it to the patient to decide about tablet dosage for pain treatment but there is also examples of when the nurse provides no tablet for sleeping problems as well as routinely interrupts pain treatment infusion with little or none consideration to the individual. The least described type of incidents is meal which include examples of opportunity to choose where and when as well as what to eat and how much. 

In the next step of analysis, positive incidents were identified as stimulating patient participation and the negative incidents as inhibiting. In Table 2, an overview of these two main areas along with patients' responses to nurses' behaviours are provided. The nurses' behaviours are illuminated using direct quotations that illustrate the connection with the narratives. 

### 3.2. Stimulating Patient Participation

#### 3.2.1. Regarded as a Person

When nurses care about patients and show a genuine interest, they feel treated and accepted as a unique person. The informants emphasised the importance of not being seen solely as an illness or a bed number. Nurses showed that they were accessible: “The nurse was there when I needed it. She was personal towards me, took her time and sat down with me.” The nurse confirmed the patient by showing “that she cared and wanted to get to know me. She could really confirm my feelings, I felt I was believed”. The fact that the nurse listened and asked questions was considered crucial: “She really listened to me and understood my situation. She asked questions to get an overall picture of my condition and find out what I like and want. Questions also mean that I have to reflect all the time. It helps me to understand my thoughts and how I can process different things.”

#### 3.2.2. Engaged through Information

When nurses provide information adapted to the patient's needs, he/she is motivated to actively participate in own care. The nurse gave the necessary explanations: “She made sure I got the information I wanted and needed. It was really good getting it from one and the same nurse. She explained what the illness meant and how it was all connected, for example, why I took this pill and was given that injection. I was given time to think and ask questions, so I know what it is all about.” The nurse also gave written material: “I was given brochures and books to read, which enabled me to form my own opinion and understand better how it is all connected. Then it was easier for us to talk about my illness and what was going to happen next.” It was considered important that the nurse acts as a mediator of contacts: “She helped me so that I got to talk with other patients about their experiences and the treatment I was going to begin on. The nurse also took me on a guided tour to say hello on the ward where I was going to be treated and see how it all works.” The informants also emphasised the importance of the nurse giving tips about self-care: “I was given tips about what to do to make it easier, how to take care of the bandage, give the injections at home, and take care of myself when it comes to food and exercise.”

#### 3.2.3. Acknowledged as Competent

When the nurse starts with and utilizes patients' own knowledge, they feel as an asset in their cooperation. The nurse discussed and made agreements: “She always included me in discussions because she needs my knowledge, said I was an expert. Nothing was done until we had had a discussion. I was involved and in control.” The nurse also handed over responsibility: “I have been allowed to decide on my pain treatment and I take the pills when I need them. That means I do not have to press the call button as soon as it hurts and then I can wait longer so that I do not get so drugged and constipated.” 

### 3.3. Inhibiting Patient Participation

#### 3.3.1. Abandoned without Backup

When a nurse, who is expected to provide support, seems to view patients in an unreflected way, they feel alone, ignored, and let down. A nurse withdrew from the patient: “I was so unhappy and she just looked at me indifferently. She must have thought that I could do that myself and was just trying to get out of it. You have to dare meet person to person.” A nurse was non-supportive during the medical ward round: “I tried to give my views during the round and didn't get any help from the nurse. She was silent and didn't dare back me up in front of the physician. They talked about me, but I wasn't asked a single question; I felt ignored and upset. It would have been better to have been backed up directly instead of her coming back afterwards and trying to put everything right.”

#### 3.3.2. Belittled Verbally

The way a nurse communicates can make patients feel depreciated. A nurse disparaged a patient with baby talk: “The nurse talked to me like I was a child; that belittles me as a person and gives an impression of insincerity.” A nurse made ironic remarks about an experience: “I was told to point at a ruler and got the answer: my dear, you can't be in that much pain. If you were, you'd be both in a cold sweat and more affected. Now, you just think about it one more time.”

#### 3.3.3. Ignored without Influence

When a nurse seems to want to exercise control and does not attach any importance to patients' views, they feel ignored and unable to participate and exert an influence. A nurse made the decisions herself and rejected the patient's views: “She took control of everything. When I said we should do like this instead, the nurse said: you don't understand this, what are you making a fuss for. She thought I was trying to correct her.” A nurse answered curtly: “I am inquisitive and the nurse only answered very briefly. I was constantly being told: we'll have to wait and see what the physician has to say. Surely, it is possible to answer one of my questions reasonably. Maybe she's not allowed to tell me, but she could at least tell me that.” A nurse neglected making notes in records: “She didn't write what I has asked to be written in my record. I think that is misconduct. When so many people are involved in my care, what is written down is important and it is often wrong; that scares me. When I read the epicrisis of the nursing care plan, I saw that they had copied the old one. It would have been good if I had been allowed to take part in the planning and evaluated my care.”

## 4. Discussion

This study, based on patients' experiences from inpatient somatic care, provided a picture of incidents, nurses' behaviours that stimulate or inhibit patients' participation and patient reactions on nurses' behaviours. The patients are in the best position to make the necessary observations and evaluations. A purposeful sample was used in order to obtain a varied picture of critical incidents of significance for patient participation. In this study, 17 inpatients provided a total of 105 critical incidents which, according to Flanagan [29], may be sufficient for a meaningful analysis. The findings are based on these informants and their ability to describe experiences of patient participation in nursing care. Although a majority of these informants were able to name some of the registered nurses on his/her ward, it is not certain that they in fact were able to distinguish “nursing” from experiences with other care providers. 

The sample included informants with different experiences, which increases the possibility of shedding light on the researched question from a variety of perspectives. Various ages, diagnoses, wards, and cultural backgrounds contributed to a rich variation which, taken as a whole, can be regarded as a strength. Actions were taken to enhance credibility in data collection. At the end of each interview, the main conclusions were verbally summarised by the interviewer and the informant supplemented, verified, and further developed the content. When 14 interviews had been conducted, earlier data were replicated and nothing new was added. The interviews were conducted and transcribed verbatim in their entirety by the same person, which enhanced the trustworthiness of the data material collected.

Credibility in the analysis was enhanced by continuously switching between the whole and the parts, and comparing and revising until a final classification emerged from the data material. Rigor was ensured by systematically handling the data, repeatedly reading, identifying, and reflecting on the critical incidents. To increase credibility, two of the authors (Inga E. Larsson and Monika J. M. Sahlsten) discussed the classifications including direct quotations in order to reduce bias which is recommended by Flanagan [29]. Finally, two researchers (Kerstin Segesten and Kaety A. E. Plos) not previously involved in the study reviewed and commented on the classifications, which included citations. 

This study is based exclusively on the patients' experiences. To provide a more complete picture, a future study may include observations of interactions between nurses, physicians, and patients but also interviews afterward to get their perspectives on why they behaved the way they did. Many factors influence each interaction, and asking why could provide more insight and knowledge. Only inpatient somatic care has been highlighted and, obviously, other patients and settings need to be explored.

 The findings reveal incidents that arise in everyday situations on a hospital ward. The incidents pinpoint situations in which nurses may risk to overstep the mark. Medical ward rounds still seem to be an incident not conducted in a democratic fashion. Patients seem to have limited opportunities to actively participate. Weber et al. [32] states that the rounds serves as a central marketplace for information where the main topic for physicians and nurses is medical information. The patients are only asked in order to reach agreement on decision-making or checking outcomes of treatment. Nursing documentation seems also to be an incident where patients have limited opportunities to exercise influence. The hierarchical nursing classification system carried out in detail may mainly serve organisational and administrative purposes [33] and therefore disregard the patients. The goal has been to record work done by nurses and to provide evidence for performed interventions. Accordingly, nursing documentation is regarded as a matter for nurses and the fact that patients also have views on its content seems to have been noticed earlier in only two studies of patient participation [4, 28]. Drug administration appears to be an incident where ward policies and protocols seem to be emphasised rather than an individual's comfort needs. Pain is an individual experience where patient participation is of uttermost importance for the recovering [34]. The most basic nursing care situations such as participation in daily living skills are not described with the exception of meals, indicating that it may be obvious and/or of minor importance. 

The findings reveal that stimulating patients' participation occurred when nurses treated the patient as a valuable coworker. This emphasises the importance of a person-centred care and of achieving a genuine connection and trusting companionship, in line with Tutton [24] and Sahlsten et al. [25]. Each patient's own capacity needs to be reinforced in order to optimise participation where patient and nurse share control and responsibility. To achieve this balance, a nurse ought to develop a personal, “ordinary”, and spontaneous approach in nursing practice. Morrison [35] states that this promotes recovery and makes patients feel good in themselves. Our findings highlight that if patients are to feel regarded as unique persons, it is crucial to break free from preconceptions and assumptions of what their needs are and enter into each patient's world. Patients need to feel that the nurse understands their situation and unique prerequisites, which is a starting point for being actively involved in one's own nursing care. 

According to the informants, it is important to become motivated and engaged through information. Information constitutes the basis of patient participation [36]. It might be helpful to think of the patient as using and trying to implement evidence-based practice, as pointed to by Edwards [37]. Patients need to find acceptable interpretations of what is happening to them, which is essential for participation. Patients collect information and take action according to their own assessment of credibility and trustworthiness of information given [4]. If different nurses appear to provide contradictory information or opinions, the patient could be confused as it means that the starting point for coping and action strategies keeps changing. Consequently, information needs to be adequate, individually adjusted, coordinated, and univocal. To meet the patients' needs, nurses have to use pedagogical strategies that promote learning such as focusing on the patient's process of reflection. This implies in-depth questions to induce patients to be self-reflective in order to utilise their own full potential in line with Sahlsten et al. [25]. 

The findings suggest that a patient, who is acknowledged as competent, presupposes stimulation and encouragement as a successful doer and owner of knowledge, in line with Hughes [38] and Tutton [24]. Patients' desire to do as much as they can by themselves may be seen as a basic human characteristic. Consequently, it is only the patient who can decide what is in his/her own best interest and nurses are then engaged in supporting. If possibilities to choose and make decisions are maximised, this may result in increased motivation to take responsibility, and exert influence and control [36, 39]. According to the informants, this leads to a sense of independence, which increases well-being, but a nurse then needs to relinquish some control, rather than exerting it.

The findings reveal that inhibiting patients' participation occurred when nurses treated patients so they felt neglected and as a helpless object of a nurse's actions. This seems to indicate that a person-centred approach is devalued in favour of a task-centred one. It also indicates a maintained traditional power imbalance where a nurse is in control. This prevents companionship, which is essential for patient participation. A nurse might have a limited understanding of professional nursing care and focus on tasks, which could result in the patient easily becoming a passive object [40]. 

When patients perceive themselves as being abandoned without backup, this indicates that nurses may use a protective mechanism to screen off emotional or advocacy aspects of their work. This may be due to working under time pressure or an idea that connecting with the patient is risky in a professional relationship. The patient is left abandoned and lonely. A patient needs genuine understanding and support. Nurses may need both practical and personal support to reduce a use of blocking behaviours to be able to work in a more responsive and effective way. In order to continuously develop self-awareness and critical monitoring skills, a professional nurse can participate in, for example, clinical nursing group supervision. This may increase nurses' ability to reflect and develop their behaviour in patient encounters. 

To provide sufficient support during medical ward rounds was surprisingly an expectation on nurses by all informants. In order to optimise patient participation, nurses need courage to back up patients to reach self-advocacy and also to be sufficiently confident to question procedures, which are to the patient's disadvantage. However, nurses can see themselves as, and acts as, an intermediary with the physician. The rounds are then perceived as “his show” which may lead to hesitation to interfere. Rounds have long been criticised for taking place with little or no patient input. Patients rarely get explanations or are encouraged to ask questions [41], an outdated routine that does not satisfy the demands of patients today. If medical ward rounds should continue in its present shape, patients need information regarding its actual aim, which seems to be to, as physician, get a face on the patient for whom the care planning is done [32]. 

When patients feel belittled verbally, a nurse may exercise the power of language or behave as a parent figure, also pointed to by Hewison [42]. This reinforces a patient's vulnerability and inhibits open communication and cooperation. The nurse disparages the patient in order to be in charge and sets the parameters for what is acceptable. McCabe [43] claims that professional nurses need to be aware of the impact the way they choose to communicate has on their patients. Communication is a powerful tool that mediates ideas, attitudes, and information, but it can also reinforce nurses' authority and hinder or exclude patients so they become increasingly dependent according to Kettunen et al. [44] or result in reluctance. 

Being ignored without influence mirrors nonrespect and no recognition of patients' requests and their right to participate. By recording the patients' views, things they regard as important will be revealed and made visible, also pointed to by Kärkkäinen and Eriksson [33]. This presupposes that the recorder knows the patient, which perhaps was not the case here. When a nurse neglects the importance of written documentation, the informants here felt that they were exposed to risks. Records can be used as working documents for both parties which may improve the content. We recommend that a nurse provide a notebook and encourage patients to keep their own notes. This could support them to remember, prepare for meetings for example, rounds, and ask questions. It can also help patients to participate and take a higher degree of control in their own care.

When nurses have a bossy or patronising attitude, this reflects a belief that it is the nurse who knows best what is in the patient's interest. This results in the patient being excluded, in line with Henderson [45]. The level of control that nurses themselves have over their practice has been shown to affect the level of active patient participation [20]. If nurses perceive themselves as diminished and not seen, they may repress patients. Empowering the patient can only be accomplished if nurses themselves are empowered [46].

## 5. Conclusions

This study, based on patients' experiences from inpatient somatic care provided a picture of incidents, nurses' behaviours that stimulate or inhibit patients' participation and patient reactions on nurses' behaviours. In order to promote patient participation, nurses need to be aware of the situations where they could overstep the mark and which of their own behaviours lead to promotion or hindrance. Our findings suggest that there is scope for developing nurses' behaviours in order to activate patients in their own nursing care. The findings may increase understanding of patient participation in nursing practise, education, policymaking, and evaluation. Further verification of the findings is recommended, either by means of replication or other studies in different settings.

## Figures and Tables

**Table 1:** Incidents and turning points based on patients' narratives of critical incidents.

Incidents*	Turning points
Medical ward round	- No support for patient input
	- No preparation ahead of medical ward round

Nursing ward round	- Genuine presence and search for patients' experience and views
	- Distance with limited support for patient input

Information session	- Meaningful and sufficient information
	- Missing, insufficient or inadequate information

Nursing documentation	- No invitation to participate
	- No recording of the patients' views

Drug administration	- Leave to patient to decide about tablet dosage for pain treatment
	- No tablet for sleeping problems
	- Interrupt pain treatment infusion by routine with little or no consideration to the individual

Meal	- Opportunity to choose where and when to serve the meal
	- Opportunity to choose what to eat and how much

*The incidents are ranked from most to least frequently described type of incident.

**Table 2:** An overview of the two main areas along with patients' responses to nurses' behaviours, based on patients' narratives of critical incidents.

Main areas	Patients' responses	Nurses' behaviours
	Regarded as a person	- Accessible - Confirms - Listens and asks
Stimulating patient participation	Engaged through information	- Gives necessary explanations - Gives written material - Acts as intermediary of contacts - Gives tips about self-care
	Acknowledged as competent	- Discusses and makes agreements - Hands over responsibility

	Abandoned without backup	- Withdraws - Nonsupportive during the medical ward round
Inhibiting patient participation	Belittled verbally	- Disparages with baby talk - Makes ironic remarks about an experience
	Ignored without influence	- Decides herself and reject views - Answers curtly - Neglects making notes in records
